# The Influence of COVID-19 on Entrepreneur's Psychological Well-Being

**DOI:** 10.3389/fpsyg.2021.823542

**Published:** 2022-01-14

**Authors:** Zhengda Xu, Heqi Jia

**Affiliations:** ^1^Business School, Beijing Technology and Business University, Beijing, China; ^2^Business School, Central University of Finance and Economics, Beijing, China

**Keywords:** COVID-19, entrepreneur, psychological well-being, firm performance, COR theory

## Abstract

This research focuses on the influence of COVID-19 on entrepreneurs' psychological well-being (PWB) in China. A start-up's performance is believed to play an important moderating role. This study uses 2 years of tracking data of 303 entrepreneurs from Shandong Providence, China. Based on conservation of resources (COR) theory, this study found that COVID-19 will significantly decrease entrepreneurs' PWB. A start-up's past performance will enhance the negative influence of COVID-19 on entrepreneurs' PWB. This study contributes to the literature on entrepreneurship, COR, and PWB. The findings can also guide entrepreneurs to maintain well-being during the pandemic and post-pandemic era.

## Introduction

The COVID-19 pandemic is not only negatively affecting the economic growth (Walmsley et al., 2020; Backer et al., 2021; Elgin et al., 2021), and business management (Andries et al., 2020; Fairlie and Fossen, 2021; Spiegel and Tookes, 2021; Verbeke and Yuan, 2021), but it is also influencing peoples' physical health (Shamim et al., 2021) and well-being (Khan et al., 2020; Ripp et al., 2020). The SMEs have suffered more from the pandemic than big companies. Based on C*oronavirus (COVID-19): SME Policy Responses*, published by the Organization for Economic Co-operation and Development, as of May 2020, 99.1% of China's big companies had resumed normal operations, whereas only 91% of small and medium-sized enterprises (SMEs) had resumed normal operations. Compared with large enterprises, SMEs had shown a weaker ability to recover (OECD, 2020).

Entrepreneurs suffer from the double impact of the pandemic. Their health is threatened by the pandemic directly. Their income decreases as their start-up firms are closed, which will further affect their well-being. According to the Enterprise Survey for Innovation and Entrepreneurship in China, entrepreneurs showed a high level of anxiety during the pandemic. For example, Rong Chao, founder of Yihua Technology (a fresh flower B2B platform company), said that Spring Festival and Valentine's Day were generally the peak seasons for the fresh flower industry, but the outbreak of the pandemic made the company lose income, which made him feel stressed and lose sleep (Peng, 2020). The vice president of Northern Light Venture Capital stated in an interview that if the companies performed and financed well before the pandemic, it would be harder for them to survive than for those with poor performance during the pandemic which made entrepreneurs stressed out (Wang, 2020). In this context, how entrepreneurs maintain well-being has become a very important topic (Patel and Rietveld, 2020).

Entrepreneurs' psychological well-being (PWB) has increasingly attracted scholars' interests (Marshall et al., 2020). Currently, researchers have already found that entrepreneurs' PWB can help them recognize opportunities (Gielnik et al., 2012) and help their firms perform better (Chao et al., 2007; Baron, 2008; Uy et al., 2017). Many studies have already analyzed the factors that affect entrepreneurs' PWB, such as entrepreneurs' prior start-up experience, active coping, moods, and motivations (Uy et al., 2013, 2017; Hahn, 2020). However, few studies have focused on entrepreneurs' PWB during the COVID-19 pandemic and the moderating role of firms' characteristics. While the organization and environment characteristics will also affect the entrepreneur's well-being.

Scholars have found people chose different coping strategies when facing difficulties under different context (Moos, 1984; Hobfoll, 2001; Uy et al., 2013). The entrepreneur's behavior would be adjusted according to the enterprise s' characteristics. Entrepreneurs are more inclined to make risky decisions in smaller enterprises, while entrepreneurs are less inclined to make risky decisions in larger enterprises (Smith et al., 1988). However, no research has focused on how the interactive effect of environmental changes and firm characteristics influence entrepreneurs' well-being.

Moreover, current research on the relationship between the pandemic and individual PWB has been relatively limited, and the impact of the pandemic on entrepreneurs' well-being particularly has been overlooked. For entrepreneurs from the same area, the pandemic will have different impact on them. Based on the prior research, we think this may be caused by their start-up's characteristics. However, the relevant impact mechanism has not been paid attention to.

Using conservation of resources (COR) theory, this paper explains how the pandemic and the characteristics of entrepreneurial companies will affect entrepreneurs' PWB. COR theory explains why people strive to obtain, retain and protect resources, and how individuals respond to threats of losing resources (Hobfoll, 1989). This theory has been used by many scholars in entrepreneurship area to predict stress response of the resource losing situation (Bonanno et al., 2007; Lanivich, 2015; Williams and Shepherd, 2016). This paper uses the data of a 2-year follow-up survey of entrepreneurs in Shandong Province, China; analyzes the impact of the severity of the pandemic in the region where the SMEs are located on the entrepreneur's PWB; and estimates the moderating effect of firm performance. This paper finds that the pandemic will significantly decrease the PWB of entrepreneurs, and firm performance strengthen the negative relationship between the pandemic and entrepreneurs' PWB.

This paper makes the following contributes. First, according to COR theory, this paper explores how resource direct loss, potential loss, and difficulty in obtaining new resources for start-ups during the pandemic decrease entrepreneurs' PWB. This enriches the existing PWB research framework. Second, this paper incorporates the organizational context into the framework of research on entrepreneurial wellbeing. This paper creatively analyzes how the characteristics of firms affect the relationship between the pandemic and entrepreneurs' well-being, which provides a new perspective for further understanding the relationship between entrepreneurs and firms. Finally, this paper makes some contributions to the COR theory. Using the samples from entrepreneurial context, this paper extending the boundary of COR theory by extending the scope of the resources list proposed by Hobfoll (2011). The research conclusion of this paper also helps entrepreneurs better understand the past and future expected performance of firms in the pandemic and post-pandemic eras. It also helps them maintain PWB and enables enterprises to achieve sustainable development.

## Theory And Hypothesis

### Conservation of Resource Theory

COR theory proposes that when an individual perceives that resources are threatened by loss, experiences the actual loss of resources, or does not obtain enough resources after making an appropriate investment in them, they will experience stress and decreased well-being (Hobfoll, 1989, 2001). The core concept of COR theory is resource. Hobfoll (1989) defined resources as valuable objects, personal characteristics, conditions, energies, or anything that can help a person gain more of the above mentioned resources. In developing of the theory, scholars have expanded the definition of resources to include anything that can help people achieve their goals (Halbesleben et al., 2014). The COR theory explains that environmental factor is an important factor threaten resource loss (Hobfoll, 1989). Environmental challenges the instrumental value and symbolic value of resources that can help people to gain more resources and define people who they are (Brown and Andrews, 1986). The dynamic and uncertain nature of the environment is the main reason that cause resource uncertainty (Adomako, 2021).

The COR theory has been widely used in many research situations, such as organizational situations, and health situations (Hobfoll, 2001). For example, some scholars used COR theory to explain how individual human capital can bring positive emotions to themselves through entrepreneurship in disaster situations (Williams and Shepherd, 2016). Scholars also used COR theory to explain entrepreneurial behavior (Lanivich, 2015) and the consequences of entrepreneurial failure (Yu et al., 2020). COR theory combines a variety of perspectives to explain the relationship between entrepreneurship and well-being, such as the value creation perspective (Brieger et al., 2021) and work-family balance perspective (Leung et al., 2020). Acquiring, protecting, and developing resources are important mechanisms in COR theory to deal with resource loss, which explains why some people can deal with resource uncertainty (Lanivich, 2015; Adomako, 2021). This theory can also help us understand how the loss of resources affects people's mental health (Hobfoll, 1989; Lanivich, 2015). Isolation, shutdown, and other activities during the pandemic greatly affect the preservation and acquisition of enterprise resources. Therefore, we can explore the internal mechanism of the impact of the pandemic on entrepreneurs' PWB using COR theory.

### COVID-19 and Entrepreneur's PWB

According to COR theory, personal psychological stress will occur when their resources are threatened with loss, their resources are actually lost, or they fail to gain sufficient resources following significant resource investment (Hobfoll, 2001). Moreover, the occurrence of negative life events often has a stronger impact on individual physiology, cognition, emotion, and social response than positive life events (Hobfoll, 2001). Entrepreneurs are more sensitive to resource loss than non-entrepreneurs (Lanivich, 2015). The entrepreneurship is a process for an entrepreneur recognizing, developing and managing resources (Corbett, 2005; Busenitz and Arthurs, 2007). The entrepreneurs' self-value is generated from entrepreneurial process (Williams and Shepherd, 2016). According to the COR theory, the broad definition of resources is anything that can help people achieve their goals (Halbesleben et al., 2014). Entrepreneurs incline to pay attention to the resources that are related to the start-ups. The resource changes will influence entrepreneurs' behavior. Scholars found that entrepreneur persistence will decrease with the potential resource loss (Holland and Shepherd, 2013).

The COVID-19 pandemic, a negative event of a wide scope and long duration, will have a strong impact on individuals (Wolfe and Patel, 2021). The pandemic is a typical resource-poor environment (Thorgren and Williams, 2020). Thus, we conduct that there are two possible mechanisms for the impact of the pandemic on entrepreneurs' PWB.

On the one hand, the pandemic has brought direct and potential losses of resources. The COVID-19 pandemic rapidly led the world to an unexpected recession (World Bank, 2020). To slow down the spread of COVID-19, many governments have taken drastic measures, such as closing borders, sealing up cities, and promulgating stay-at-home restrictions and social distancing policies (Kuckertz et al., 2020; Han et al., 2021; Tang et al., 2021). These policies prevent the process of resources development. The market demand had decreased significantly because consumers were unable to shop in the stores (Fairlie and Fossen, 2021), this caused the direct loss of the resource of the firm. The fixed cost still exists after the shutdown of enterprises, but firms are unable to produce and obtain profits, which has had a serious impact on global entrepreneurial activities (Björklund et al., 2020; Brown and Rocha, 2020; Patel and Rietveld, 2020). These challenges hinder entrepreneurs from achieving their goals. According to the COR theory, entrepreneurs will regard these as the loss or potential loss of resources, so their PWB will be reduced.

The pandemic has reduced the value that human capital can provide (Yarovaya et al., 2021), making it difficult for enterprises to respond and adjust effectively to emergencies. Human resource is one of the most important resources for start-ups. It is also the carrier of the knowledge resource (Halbesleben et al., 2014). The loss of human resource not only influence the operation of the firms directly but also influence the ability and efficacy for start-ups to obtain and explore new resource (Williams and Shepherd, 2016). The pandemic led to the inability of some employees to work on duty, and the low efficiency of remote work decreased the performance of employees (Kumar et al., 2021; Wang et al., 2021). These factors have further reduced the income of enterprises. The decrease of enterprise income will lead to a series of layoffs, which will further reduce the human resources of the enterprise (Butterick and Charlwood, 2021). Even if the pandemic has been temporarily controlled in some areas, the possibility of recurrence will also bring potential losses to enterprises. Once the pandemic reappears, enterprises will face shutdown again. Therefore, in areas with serious pandemic recurrence, SMEs face greater potential resource losses. According to the COR theory, direct loss and potential loss of resources will reduce the well-being of entrepreneurs. Therefore, the more serious the pandemic, the more resource losses of the firm, the lower the PWB of entrepreneurs.

On the other hand, the pandemic has increased the difficulty of obtaining new resources, making entrepreneurs unable to obtain sufficient resources even they make a great effort. Scholars have found that the pandemic has significantly reduced the total capital in the Chinese market, increased the scarcity of market resources, and made it difficult for entrepreneurs to obtain investment by making the effort (Brown and Rocha, 2020). The pandemic also causes entrepreneurs to make efforts more difficult. During the pandemic period, many countries have promulgated stay-at-home restrictions (Kuckertz et al., 2020; Tang et al., 2021), which has made it impossible for entrepreneurs to take the initiative to find and obtain resources. The pandemic has also reduced the trust behavior of the overall market (Li et al., 2021) and has increased the cost of resource acquisition. According to the COR theory, entrepreneurs' well-being will decrease when they cannot obtain resources through effort. The more serious the pandemic becomes, the less likely entrepreneurs will be to obtain new resources, and the lower will be the PWB of entrepreneurs. Based on this, we proposed the following hypothesis:

H1: Covid-19 pandemic will decrease entrepreneurs' PWB.

### The Moderating Effects of Firm Performance on the Influence of Pandemic on PWB

The performance of start-ups will affect the relationship between the pandemic and entrepreneurs' PWB. An enterprise's performance is closely related to its resources (Bharadwaj, 2000; Beleska-Spasova et al., 2012). According to resource-based view (RBV), resources are necessary for the operation of enterprises (Alvarez and Busenitz, 2007). If an enterprise wants to perform well, it must acquire a large amount of resource investment, such as human resources (Hitt et al., 2001), technical resources (Powers and McDougall, 2005), or all kinds of capital (Quas et al., 2021). Based on RBV and COR theory, there may be two mechanisms for the impact of the performance of SMEs on the relationship between the pandemic and entrepreneurs' PWB.

First, the good past performance of firms will increase entrepreneurs' expectations of available resources in the future. Managers will infer the future performance of firms based on their past performance and make corresponding decisions (Lages et al., 2008, 2013; Hadad et al., 2013). Therefore, when the past performance of SMEs is good, entrepreneurs will expect to obtain more benefits and resources in the future. During the pandemic, the governments issued many shutdown policies, and enterprises were unable to continuously obtain resources from outside (Brown and Rocha, 2020), which makes it difficult to realize the business expectations of entrepreneurs. The better the past performance, the bigger the gap between entrepreneurs' expectations and reality. According to COR theory, entrepreneurs show lower PWB when they perceive greater potential resources loss. On the contrary, if the previous performance is poor, the entrepreneurs' expectations for the future growth of the enterprise is relatively low. The potential future loss perceived by entrepreneurs during the pandemic is also low. Therefore, the negative impact of the pandemic on entrepreneurs' PWB is weaker when the past performance is bad.

Second, when the past performance of the firm is good, the pandemic will cause more resource loss directly. Thus, the good past performance increases the negative impact of the pandemic on entrepreneurs' PWB. According to RBV, good performance means that the SMEs had invested more resources in the early stage (Mishra and Zachary, 2013; Choi et al., 2021), and the SMEs had accumulated more resources at present. To ensure the sustainable competitive advantage and exploit the potential opportunities in the future, enterprises with good performance will continue to invest more resources to ensure a good growth rate (Phillips and Kirchhoff, 1989), such as continuously looking for venture capital (Chittenden et al., 1996). When the pandemic occurs, no matter how much the resource of start-ups has invested before, the expected income they can obtain will be reduced to a very low level. This makes the enterprises with better performance face greater losses, so the PWB of entrepreneurs will be worse.

Based on these analyses, this paper puts forward the following hypothesis:

H2: Firm performance will moderate the relationship between the COVID-19 pandemic and entrepreneurs' PWB. Specifically, the better the performance of the firm, the greater the negative impact of the pandemic on the entrepreneurs' PWB.

**Figure 1:**
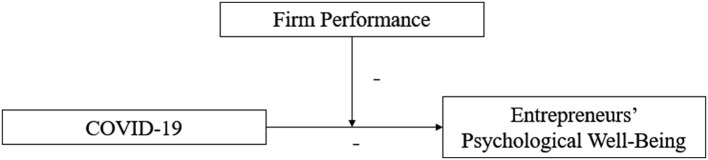
Theoretical model.

This study summarizes the hypotheses of the present study in Figure 1.

## Methods

### Sample

This study conducted a two-wave survey by questionnaire to verify the hypotheses. This study selects only one province for collecting data to reduce the impact of macro-environmental factors, such as different entrepreneurial policies, regional epidemic severity, and policy differences during the epidemic that affect entrepreneurs. According to the Statistical Yearbook of the National Bureau of Statistics of China in 2020, Shandong province ranked the first in China in terms of the number of self-employed by the end of 2019. Shandong province is one of the most active entrepreneurial areas in China. Therefore, it is appropriate to use the SMEs in Shandong province for the sample of this study.

This study first collected the data in November 2019. In the first survey, we obtained 305 founder-CEOs' basic information (e.g. gender, age, education, marriage, and entrepreneurial experience). We also asked the entrepreneur to introduce one top management team member to finish another questionnaire to avoid the common method bias (Chin et al., 2021). This study collected basic firm information from this manager (e.g., firm age, firm size, firm performance, and industry). In March 2020, we tracked these founder-CEOs and managers to collect the dependent variable and moderate variable. We obtained 303 valid samples in 2020. Thus, the final sample in this study is 303.

Among these 303 business owners, there are 215 male entrepreneurs (70.96%) and 88 female entrepreneurs (29.04%). The age of entrepreneurs is concentrated between 31 and 40 years old, with the largest proportion at 54.1%. As for the educational background, most of the entrepreneurs graduated from high school or technical high school (34.32%) and junior college (35.97%). In addition, 276 entrepreneurs are married, and 202 entrepreneurs have prior start-up experience.

Among these 303 start-ups, the most of them were established within 3 years (97.36%). The assist size of the start-ups was under 1 million RMB (90.42%). In addition, there are 74 start-ups in wholesale and retail industry (24.42%), 68 start-ups in resident services and other services industry (22.44%), and 55 start-ups in accommodation and catering industry (18.15%).

### Measurement

This study presents the measurement items for psychological well-being and firm performance in Table 1.

**Table 1:** Measurement items and reliabilities.

**Variables**	**Items**	**Alpha**	**CMIN/DF**	**CFI**	**GFI**	**RMSEA**	**AVE**	**CR**
Psychological well-being	Have you been feeling not perfectly well or not in good health?	0.756	3.573	0.924	0.921	0.092	0.905	0.445
	Been felling run down and out of sorts?							
	Have you felt that you are ill?							
	Felt constantly under strain?							
	Been getting edgy and bad-tempered?							
	Been getting scared or panicky for no good reason?							
	Felt that you are playing a useful part in things?							
	Felt capable of making decisions about things?							
	Felt on the whole you are doing things well?							
	Been thinking of yourself as a worthless person?							
	Felt that life is entirely hopeless?							
	Felt that life isn't worth living?							
Firm performance (in 2019)	Sales growth	0.903	1.412	0.995	0.983	0.037	0.825	0.402
	Market share growth							
	Net profit margin							
	Return on equity							
	Return on assets							
	Return on sales							
	Profit growth							
Firm performance (in 2020)	Sales growth	0.905	2.402	0.983	0.969	0.068	0.831	0.416
	Market share growth							
	Net profit margin							
	Return on equity							
	Return on assets							
	Return on sales							
	Profit growth							

#### COVID-19

This study used the number of confirmed cases of the coronavirus in the city where a given company is located to measure the impact of COVID-19. The greater the number of confirmed cases, the larger the impact of the pandemic. This study used the real-time statistic of the pandemic on Sina News (one of the largest news websites in China) to collect the cumulative number of confirmed cases in the city where a given company is located before March 1, 2020.^1^ The spread of the coronavirus has been effectively controlled in China since the end of February 2020. The number of confirmed cases has rarely increased since March 1st, 2020.

#### Psychological Well-Being

This study used the General Health Questionnaire developed by Goldberg and Hillier (1979) to measure entrepreneurs' PWB. This measurement has been widely used in entrepreneurship studies (Uy et al., 2013, 2017; Hahn, 2020; Marshall et al., 2020). Participants were required to respond to questions based on the situation in the past few weeks. This scale includes 12 items, such as the following: “Have you been feeling not perfectly well or not in good health?” “Have you felt constantly under strain?” The participants answered on a 4-point scale, with 1 standing for “not at all, and 4 standing for “much more than usual.” The Cronbach's alpha value is 0.756, which means the reliability is acceptable. In addition, the results (CMIN/DF = 3.573, CFI = 0.924, GFI = 0.921, RMSEA = 0.092) of the confirmatory factor analysis prove that the validity is acceptable.

#### Firm Performance

This study used the firm performance growth rate to measure firm performance (Zhao et al., 2010). Using growth rate can avoid the performance biases caused by firm size and firm industry. We used the multidimensional construct of performance in 2020 minus the multidimensional construct of performance in 2019, then we divide the multidimensional construct of performance in 2019 to calculate the firm growth rate. The larger the value, the better the firm performance.

The multidimensional construct of firm performance was measured by a 7-item scale (Stam et al., 2014; Rauch and Hatak, 2016; e.g., sales growth, market share growth). In 2019, we asked the manager, “How is the competition status for your company compared with a major competitor in the same industry?” The participants answered on a 5-point scale, with 1 standing for “falling far behind” and 5 standing for “stronger than competitors.” The Cronbach's alpha value is 0.903, which means the reliability is acceptable. In addition, the results (CMIN/DF = 1.412, CFI = 0.995, GFI = 0.983, RMSEA = 0.037) of the confirmatory factor analysis prove that the validity is acceptable.

The measurement of firm performance in 2020 is the same as the in 2019. The alpha reliability of the performance in 2020 is 0.905, which means the reliability is acceptable. In addition, the results (CMIN/DF = 2.402, CFI = 0.983, GFI = 0.969, RMSEA = 0.068) of the confirmatory factor analysis prove that the validity is acceptable.

#### Controls

Following the previous research, this study controls some individual-level and firm-level variables that can affect entrepreneurs' PWB (Uy et al., 2013, 2017; Hahn, 2020).

At the individual level, this study controlled for entrepreneurs' gender (1 = “male” and 0 = “female”), age, and education (1 = “primary school,” 2 = “secondary school,” 3 = “high school/technical high school” 4 = “junior college,” 5 = “bachelor,” 6 = “master,” 7 = “doctoral”). Marriage status was measured by a dummy variable. We coded 1 for “married” and 0 for “unmarried or divorced”. The entrepreneurial experience was measured by whether the entrepreneurs had established a firm before the current business.

At the firm level, this study controlled for the firm age, firm size, and firm industry. We asked the manager about which year the company was established. Then, we used 2020 minus the established year to obtain the firm age. Firm size was measured by the natural logarithm of the total firm assets (Tian et al., 2020). This article formed three dummy industrial variables to control, including wholesale and retail (Ind1; 1 = “Yes” and 0 = “No”), resident services and other services (Ind2; 1 = “Yes” and 0 = “No”), and accommodation and catering (Ind3; 1 = “Yes” and 0 = “No”).

## Results

### Descriptive Statistics and Correlation Analysis

This study shows the descriptive statistics and correlation coefficients for all variables in Table 2. We see that the correlation coefficient that is higher than 0.1 is significant. The absolute value of the correlation coefficient is < 0.7, which means there is no multicollinearity. The significant and negative correlation between COVID-19 and PWB (r = −0.287, *p* < 0.01) indicates that COVID-19 would influence PWB negatively. The significant and positive correlation between firm performance and PWB (r = 0.255, p < 0.01) indicates that firm performance would influence PWB positively.

**Table 2:** Mean, standard deviation, and correlation of study variable.

**Variables**	**Mean**	**SD**	**1**	**2**	**3**	**4**	**5**	**6**	**7**
1. Gender	0.710	0.455	1.000						
2. Age	38.931	6.289	0.141^*^	1.000					
3. Education	3.818	0.901	−0.032	−0.200^**^	1.000				
4. Marriage	0.911	0.285	0.055	0.334^**^	−0.128^*^	1.000			
5. Experience	0.884	0.787	−0.039	0.010	−0.142^*^	−0.120^*^	1.000		
6. Firm Age	2.611	1.107	−0.068	0.077	−0.011	−0.058	0.055	1.000	
7. Firm size	12.867	0.989	0.017	0.188^**^	0.303^**^	0.046	0.043	0.102	1.000
8. Ind1	0.244	0.430	0.025	−0.057	−0.218^**^	0.097	0.044	0.020	0.006
9. Ind2	0.224	0.418	−0.057	0.036	−0.015	−0.054	0.019	0.003	−0.090
10. Ind3	0.182	0.386	0.037	0.058	−0.314^**^	−0.003	0.026	0.003	−0.197^**^
11. COVID-19	98.290	95.874	−0.048	−0.213^**^	0.121^*^	−0.421^**^	0.104	0.114^*^	0.052
12. Firm performance	−0.222	0.211	0.049	−0.096	0.096	0.013	−0.014	0.017	0.150^**^
13. PWB	3.613	0.495	0.069	−0.164^**^	0.169^**^	0.077	−0.217^**^	0.035	−0.023
**Variables**		**Mean**	**SD**	**8**	**9**	**10**	**11**	**12**	**13**
1. Gender		0.710	0.455						
2. Age		38.931	6.289						
3. Education		3.818	0.901						
4. Marriage		0.911	0.285						
5. Experience		0.884	0.787						
6. Firm age		2.611	1.107						
7. Firm size		12.867	0.989						
8. Ind1		0.244	0.430	1.000					
9. Ind2		0.224	0.418	−0.306^**^	1.000				
10. Ind3		0.182	0.386	−0.268^**^	−0.253^**^	1.000			
11. COVID-19		98.290	95.874	−0.057	0.085	−0.008	1.000		
12. Firm performance		−0.222	0.211	0.058	−0.034	−0.125^*^	−0.153^**^	1.000	
13. PWB		3.613	0.495	0.028	−0.076	−0.172^**^	−0.287^**^	0.255^**^	1.000

*N = 303*.

** *p < 0.01*.

* *< 0.05*.

### Regression Analysis

**Figure 2:**
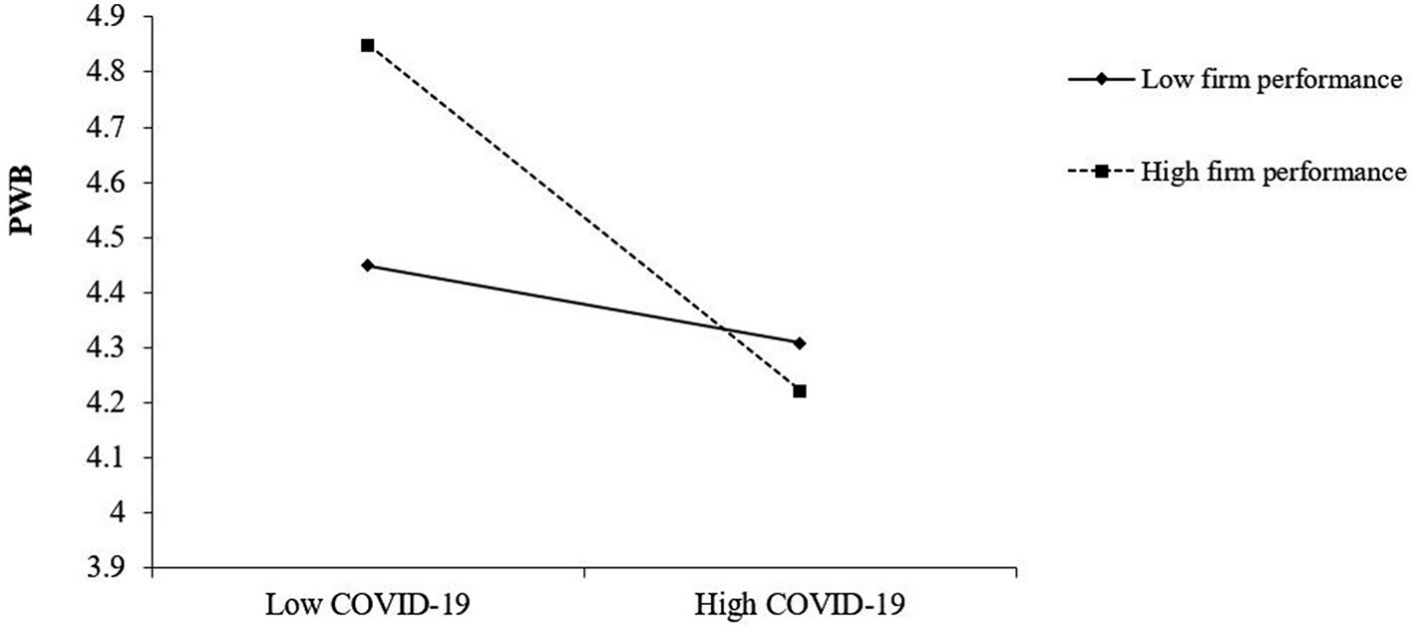
The interaction between COVID-19 and firm performance on PWB.

This study uses hierarchical regression analysis to verify the relationship between COVID-19 and entrepreneurs' PWB, and the moderate effect of firm performance. The results are shown in Table 3. The largest value of the variance inflation factor of all variables is 1.653, which is lower than 2 and thus indicates that the variables have no multicollinearity problem. The results in Model 2 show that COVID-19 has a significant negative impact on PWB (b = −0.002, *p* = 0.000). Thus, hypothesis 1 is supported. The results in Model 3 show that the interaction between COVID-19 and firm performance has a significant effect on PWB (b = −0.006, *p* = 0.000). Thus, hypothesis 2 is supported. This study depicted the interaction graph in Figure 2.

**Table 3:** Results of regression analysis.

	**PWB**		
	**Model 1**	**Model 2**	**Model 3**
Gender	0.100^†^	0.088	0.076
	(1.672)	(1.560)	(1.392)
Age	−0.015^**^	−0.015^**^	−0.013^**^
	(−2.999)	(−3.261)	(−2.933)
Education	0.035	0.046	0.061^†^
	(0.952)	(1.327)	(1.778)
Marriage	0.222^*^	0.023	0.033
	(2.177)	(0.222)	(0.329)
Experience	−0.111^**^	−0.100^**^	−0.095^**^
	(−3.183)	(−3.040)	(−2.972)
Firm age	0.037	0.047^*^	0.046^*^
	(1.505)	(2.034)	(2.036)
Firm size	−0.034	−0.035	−0.050^†^
	(−1.130)	(−1.224)	(−1.761)
Ind1	−0.084	−0.075	−0.029
	(−1.079)	(−1.025)	(−0.405)
Ind2	−0.159^*^	−0.118	−0.090
	(−2.095)	(−1.647)	(−1.287)
Ind3	−0.264^**^	−0.220^**^	−0.175^*^
	(−3.000)	(−2.648)	(−2.156)
COVID-19		−0.002^***^	−0.002^***^
		(−5.012)	(−5.471)
Firm performance		0.386^**^	0.373^**^
		(−3.106)	(3.088)
COVID-19 ^*^ firm performance			−0.006^***^
			(−4.345)
Constant	4.318^***^	4.444^***^	4.457^***^
	(10.769)	(11.779)	(12.171)
R^2^	0.144	0.253	0.299
ΔR^2^	0.144	0.109	0.046
F-Statistic	4.907^***^	8.188^***^	9.477^***^

*N = 303*.

† *p < 0.1*,

* *p < 0.05*,

** *p < 0.01*,

*** *p < 0.001*.

*Numbers in brackets refer to t-value*.

### Robustness Test

To ensure the robustness of the model, this study used an alternative moderator variable and dependent variable to rerun the hierarchical regression analysis. The results are shown in Table 4.

**Table 4:** Regressive results of robustness test.

	**Need for recovery**		**PWB**	
	**Model4**	**Model5**	**Model6**	**Model7**
Gender	−0.005	0.001	0.084	0.077
	(−0.080)	(0.02)	(1.472)	(1.370)
Age	0.006	0.005	−0.017^***^	−0.016^**^
	(1.036)	(0.843)	(−3.600)	(−3.520)
Education	0.000	−0.008	0.050	0.052
	(0.005)	(−0.192)	(1.429)	(1.477)
Marriage	−0.092	−0.098	0.022	0.013
	(−0.740)	(−0.791)	(0.211)	(0.125)
Experience	−0.006	−0.009	−0.092^**^	−0.096^**^
	(−0.155)	(−0.230)	(−2.766)	(−2.902)
Firm Age	−0.002	−0.001	0.050^*^	0.048^*^
	(−0.058)	(−0.031)	(2.150)	(2.063)
Firm size	0.088^*^	0.097^**^	−0.015	−0.006
	(−2.521)	(2.757)	(−0.531)	(−0.214)
Ind1	−0.038	−0.065	−0.073	−0.063
	(−0.424)	(−0.722)	(−0.992)	(−0.867)
Ind2	0.046	0.029	−0.133^†^	−0.125^†^
	(0.526)	(0.337)	(−1.839)	(−1.739)
Ind3	0.010	−0.016	−0.224^**^	−0.204^*^
	(0.102)	(−0.157)	(−2.674)	(−2.450)
COVID-19	0.003^***^	0.003^***^	−0.002^***^	−0.002^***^
	(9.269)	(−9.445)	(−5.920)	(−5.343)
Firm performance	0.103	0.110		
	(0.676)	(0.732)		
COVID-19 ^*^ firm performance		0.003^*^		
		(2.043)		
Employee growth rate			0.378^*^	0.479^*^
			(2.078)	(2.610)
COVID-19 ^*^ employee growth rate				−0.005^**^
				(−2.777)
Constant	1.346^**^	1.338^**^	4.226^***^	4.108^***^
	(2.926)	(−2.925)	(11.030)	(10.779)
R2	0.309	0.319	0.240	0.259
F-Statistic	10.824^***^	10.422^***^	7.613^***^	7.783^***^

*N = 303*.

† *p < 0.1*,

* *p < 0.05*,

** *p < 0.01*,

*** *p < 0.001*.

This study used the need for recovery to alter PWB. The literature about well-being and recovery are overlapped (Leamy et al., 2011). Need for recovery refers to a mental state in which an individual desperately wants to temporarily recover from job exhaustion. It is a feeling of pursuing quietness for a period (Sonnentag and Zijlstra, 2006; Sonnentag et al., 2010; Kinnunen et al., 2011). Scholars used need for recovery as an indicator of individuals' well-being (Kinnunen et al., 2011; Mache et al., 2020). Thus, we used need for recovery as an alternative variable of PWB. We used a 11-item need for recovery scale adopted from Veldhoven and Broersen (2003; e.g., “I find it difficult to relax at the end of a working day”). The items for this scale are measured on a 5-point scale from 1 (“I do not agree at all”) to 5 (“I fully agree”). The higher the score, the higher the entrepreneurs need for recovery. When entrepreneurs need for recovery means they are not in a good psychological well-being status. The results in Model 4 show that COVID-19 has a significant positive effect on the need for recovery (b = 0.003, *p* = 0.000). Thus, hypothesis 1 is supported. The results in Model 5 show that the interaction between COVID-19 and firm performance has a significant effect on the need for recovery (b = 0.003, *p* = 0.042). Thus, hypothesis 2 is supported. The results are robust.

Then, this study used growth in the number of employees as an alternative measurement of the moderator variable. We asked the participates about the growth in the number of employees of the current companies. The results in Model 6 show that COVID-19 has a significant negative effect on PWB (b = −0.002, *p* = 0.000). Thus, hypothesis 1 is supported. The results in Model 7 show that the interaction between COVID-19 and firm performance growth has a significant effect on PWB (b = −0.005, *p* = 0.006). Thus, hypothesis 2 is supported. The results are robust.

These results show that the research model is robust.

## Conclusions And Discussions

### Conclusions

According to the results of empirical analysis, this paper finds that the pandemic will significantly decrease the PWB of entrepreneurs. Firm performance will moderate the relationship between the pandemic and the entrepreneur's PWB. Specifically, the better the performance of firm, the greater the negative impact of the pandemic on entrepreneurs' PWB.

### Theorical Contributions

First, this paper explores the influence of the pandemic on entrepreneurs' PWB. Although many scholars have studied the impact of the pandemic on individual well-being in different occupational types (e.g., female health professionals; Shahbaz et al., 2021), few scholars have paid attention to the relationship between the pandemic and entrepreneurs' well-being. The pandemic has had an important impact on enterprises, especially on SMEs (Andries et al., 2020; Nummela et al., 2020; Shepherd, 2020). The dual roles of entrepreneurs made them more vulnerable during the pandemic, which will significantly influence their PWB. Based on this logic, this paper explores the impact of the pandemic on entrepreneurs' PWB and fills the gap in the research about the COVID-19 pandemic and entrepreneurs' well-being.

Second, this paper establishes a research framework about the influence of enterprise characteristics on the relationship between pandemic and entrepreneurs' well-being, which has certain theoretical contributions. Previous studies have only addressed some of the factors affecting entrepreneurs' PWB (Stephan, 2018), and have not addressed context variables. The influence of external factors on the entrepreneur's well-being changes in different contexts (Moos, 1984; Hobfoll, 2001). This paper considers the entrepreneur's startup as the most important organizational context and analyze how pandemic affects the entrepreneur PWB differently in different contexts. This paper changes the previous well-being research model from the perspective of the dynamic interaction of environment, SMEs performance, and the entrepreneurs' PWB. This perspective has certain theoretical contributions.

Finally, this paper enriches the boundary of COR theory. Based on COR theory, this paper explains the impact of SMEs' resource gain and loss on entrepreneurs' own PWB in the face of the pandemic. Due to the specificity of entrepreneurial activities, entrepreneurs focus on the resources at both individual level and firm level. Entrepreneurship is continuous process of resource acquisition, utilization and exploitation (Corbett, 2005; Busenitz and Arthurs, 2007), which this relies heavily on the entrepreneur's own ability to recognize, develop and manage resources (Hindle, 2011; Moroz and Hindle, 2012). These characteristics of entrepreneurial activity make the loss of resources in the start-ups affect the wellbeing of the entrepreneur. This study makes a theoretical contribution by extending the scope of resources in COR theory and enriching the application context of the theory.

### Practical Contributions

First, this paper can help entrepreneurs maintain wellbeing in the pandemic and post-pandemic eras. The continuous and repeated outbreak of the pandemic will not only directly affect the personal health and well-being of entrepreneurs but will cause firm resource loss and will increase the difficulty of new resource acquisition. The conclusion of this paper provides a theoretical basis for entrepreneurs to better understand resource loss and resource shortage during the pandemic, which will help them maintain PWB.

Second, this paper can help entrepreneurs better understand the relationship between enterprise characteristics and their own well-being. According to the empirical conclusion of this paper, firm performance will affect the PWB of entrepreneurs. To maintain their PWB, entrepreneurs should selectively control the development speed of their enterprises when dealing with different environmental context. For example, when predicting the lack or shortage of resources in the future environment, entrepreneurs should slow down the growth of firms to obtain better PWB.

Finally, this paper provides a certain reference for local governments to adopt relevant policies. According to the conclusion, the pandemic will significantly reduce entrepreneurs' PWB, and the negative impact is greater when the firm performance is better. Local governments should focus on helping companies with better performance in the past when formulating related support policies. These kinds of policies can decrease the impact of the pandemic on entrepreneurs' PWB.

### Limitations and Prospects

This article mainly uses samples of entrepreneurs and entrepreneurial companies in Shandong Province, China. Future research should add samples from different nations to test whether the research conclusions can be extended to other countries. Second, this paper primarily used questionnaire survey methods to verify the research questions. In the future, we can use interviews and other qualitative research methods to explore the impact mechanism of the pandemic on entrepreneurs' PWB. Finally, there are many ways to measure wellbeing. In the future, research on the impact of the pandemic on entrepreneurs' other types of well-being can be constructed.

## Data Availability Statement

The raw data supporting the conclusions of this article will be made available by the authors, without undue reservation.

## Author Contributions

ZX made the theoretical design of this article, reviewed, and revised the manuscript. HJ made the data analysis and drafted the manuscript. Both authors contributed to the article and approved the submitted version.

## Funding

This work was supported by Startup Fund Project of Beijing Technology and Business University.

## Conflict of Interest

The authors declare that the research was conducted in the absence of any commercial or financial relationships that could be construed as a potential conflict of interest.

## Publisher's Note

All claims expressed in this article are solely those of the authors and do not necessarily represent those of their affiliated organizations, or those of the publisher, the editors and the reviewers. Any product that may be evaluated in this article, or claim that may be made by its manufacturer, is not guaranteed or endorsed by the publisher.

## Footnotes

^1^Data source: https://news.sina.cn/project/fy2020/yq_province.shtml?city=CN37010000000000.
